# Supplementary material for: Imprint of urbanization on snow precipitation over the continental USA
Source: Nat Commun. 2024 Mar 15;15:2348. doi: 10.1038/s41467-024-46699-6 (PMC10943213; doi:10.1038/s41467-024-46699-6)
Supplement: Supplementary file 1 — Supplementary Information [file 41467_2024_46699_MOESM1_ESM.pdf]

Supplementary information for

Imprint of Urbanization on Snow Precipitation over the Continental USA

Kaustubh Anil Salvi[1], Mukesh Kumar[1]

Department of Civil Construction & Environmental Engineering, University of Alabama, P.O. Box 870205, Tuscaloosa, Alabama, 35487-0205

Mukesh Kumar,
Associate Professor of Civil, Construction and Environmental Engineering,
The University of Alabama,
Email: mkumar4@eng.ua.edu

Keywords: Precipitation phase, Snow to rain transition, Land-atmosphere exchange, Urbanization impacts, Climate change impacts

Content

- Supplementary figures
- Supplementary tables

Supplementary figures

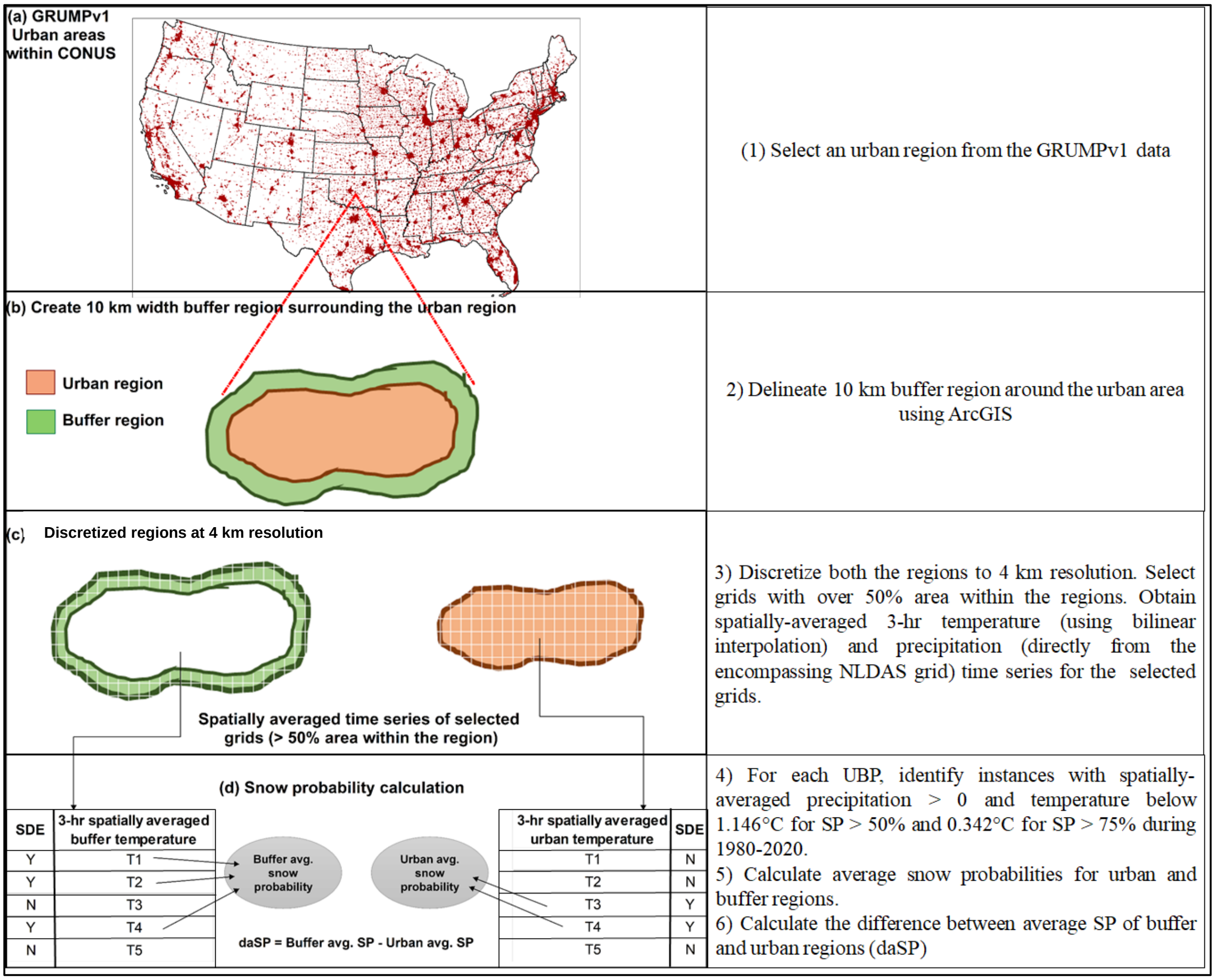

Figure S1: Approach used to obtain representative temperature and precipitation time series for urban and buffer regions and for calculating difference between average snow probabilities during 1980-2020 (daSP).

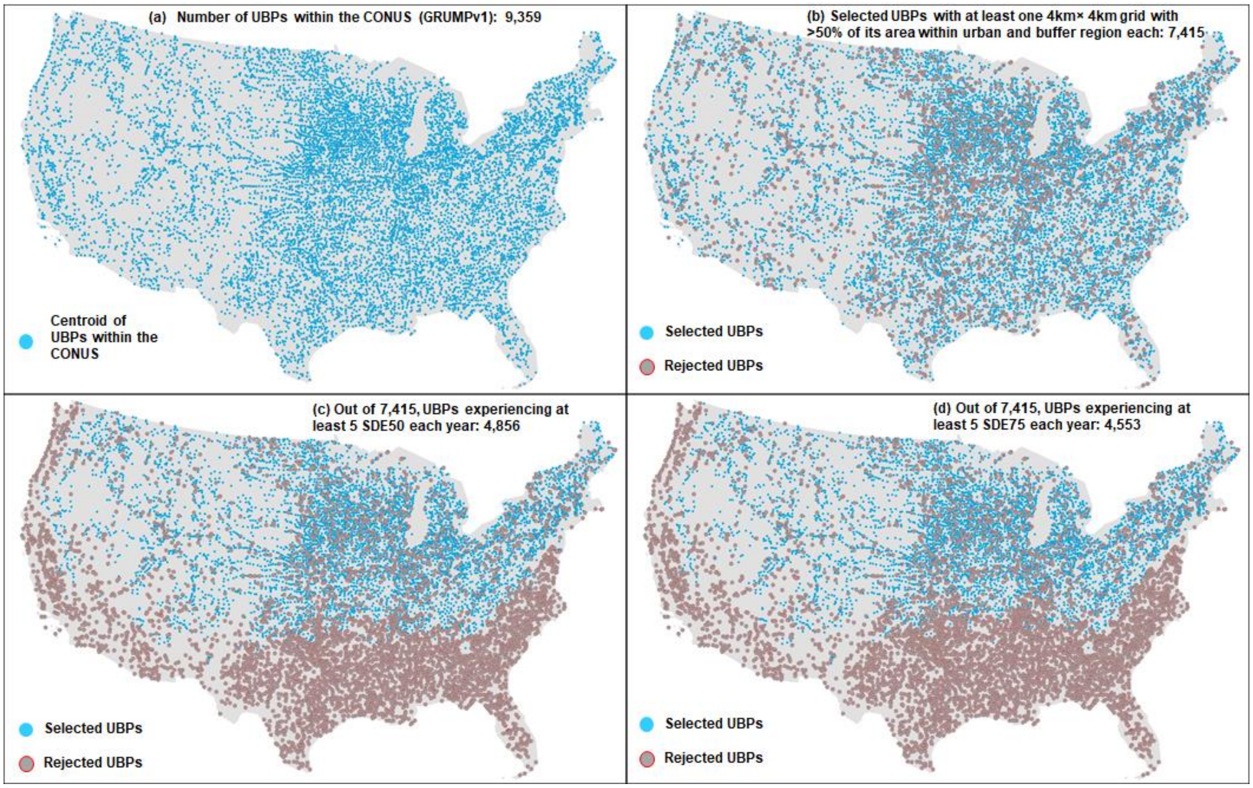

Figure S2: Spatial distribution and count of UBPs within CONUS based on GRUMPv1 data (a), selected based on the 50% grid overlap criterion (b), selected based on minimum number of SDE50 (c) and SDE75 (d) events along with the criterion outlined in (b). Source data are provided in Source_data.xlsx, on the sheet called Figure_S2.

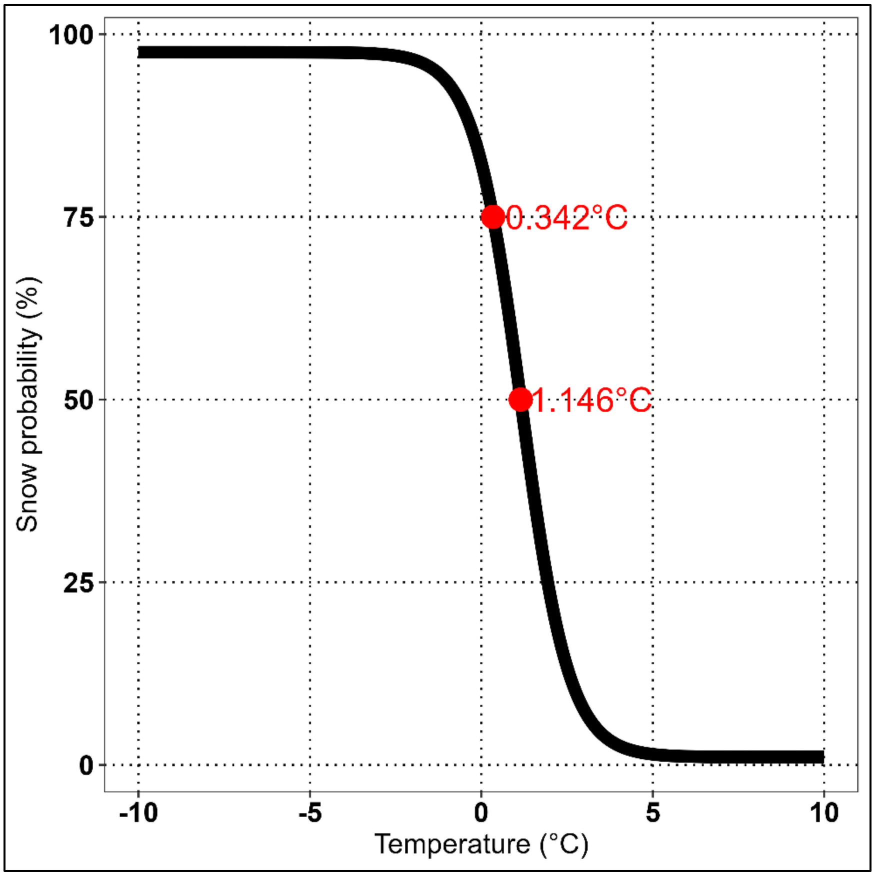

Figure S3: Variation in snow probability with temperature based on the hyperbolic tangent curve[1] ('Dai' formula.

Two temperature thresholds viz. 1.146°C and 0.342°C (which correspond to 50% and 75% of snow probabilities) are considered in this analysis. The range between -4°C to 4°C may be considered as transition zone, where relatively rapid variation in SP with temperature is observed.

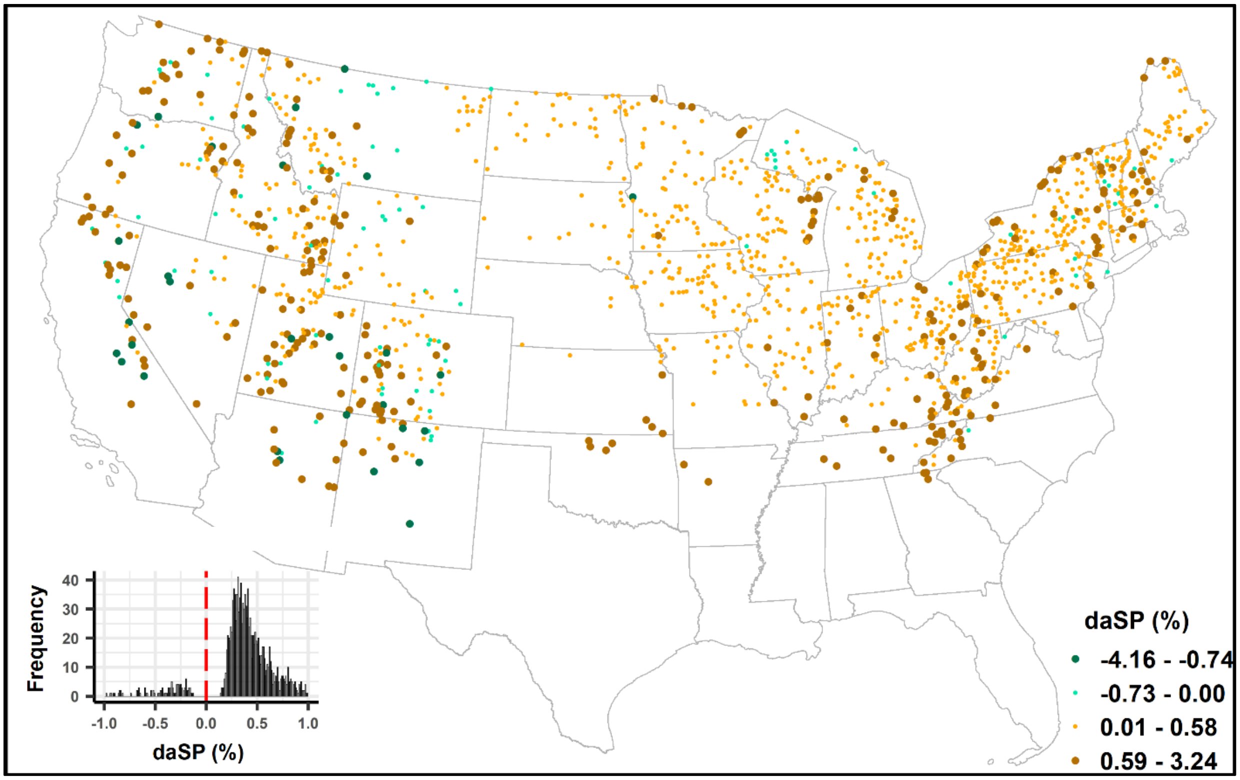

Figure S4: Spatial distribution of UBPs with statistically significant (s.s.) daSP (SDE50). 91% (1,267) of the UBPs with s.s. differences (1,387) show positive daSP (inset plot) indicating that a high percentage of buffer regions have higher average SP of SDEs as compared to their urban counterparts. Source data are provided in Source_data.xlsx, on the sheet called Figure_S4.

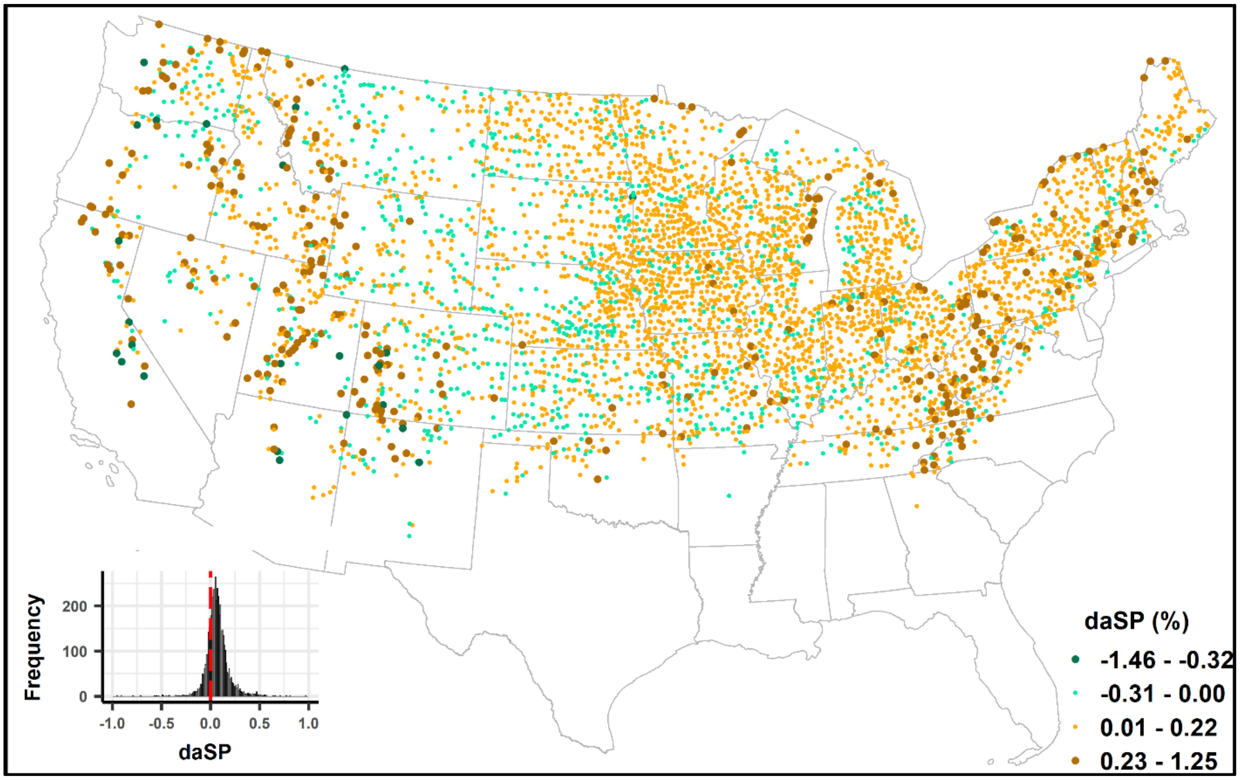

Figure S5: Similar to Figure 1, but for SDE75

Around 79% of buffer regions (3,578 out of 4,553) show higher average SP compared to their urban counterparts. Source data are provided in Source_data.xlsx, on the sheet called Figure_S5.

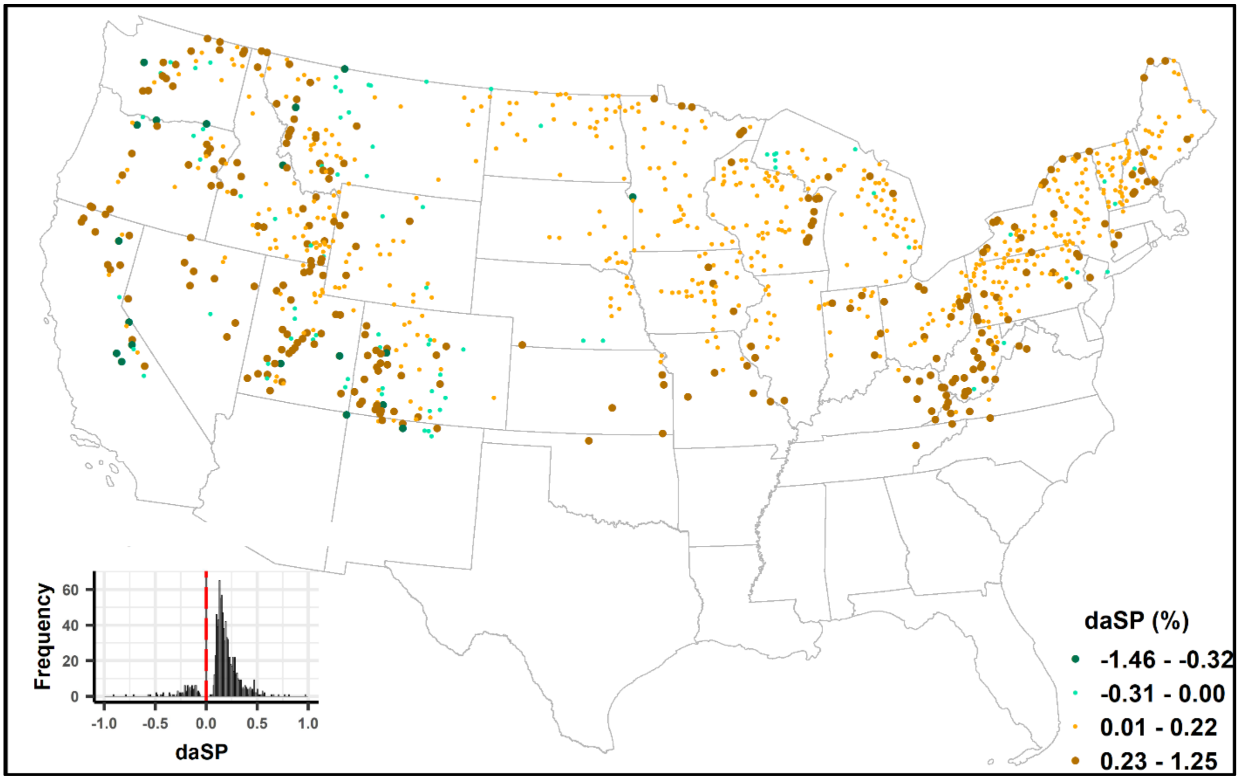

Figure S6: Similar to Figure S4, but for SDE75

Around 84% of buffer regions (851 out of 1,013) show higher average SP compared to their urban counterparts. Source data are provided in Source_data.xlsx, on the sheet called Figure_S6.

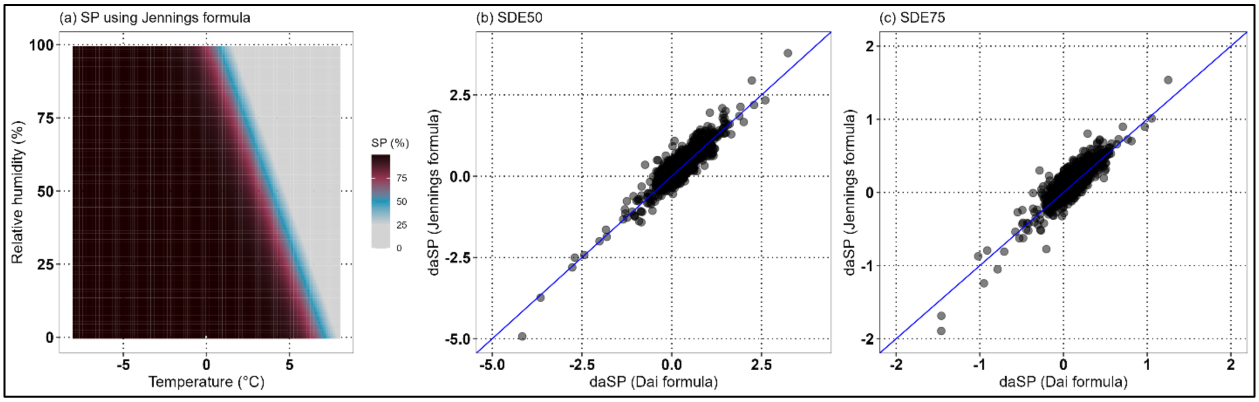

Figure S7: SP variation with temperature and relative humidity calculated using Jennings formula (a), comparison of daSP for SDE50 (b) and SDE75 (c) obtained with Dai and Jennings formulae. Irrespective of SP calculation method, the magnitude and spatial distribution of daSP obtained using both the equations exhibit strong linear association (r = 0.9 for SDE50 (b), r = 0.86 for SDE75 (c)). Source data are provided in Source_data.xlsx, on the sheet called Figure_S7.

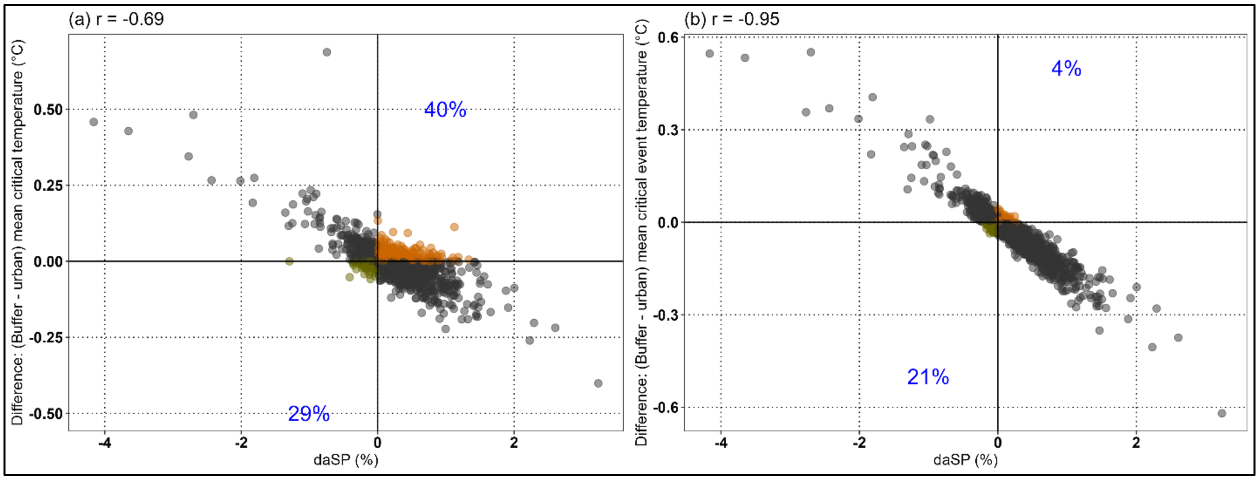

Figure S8: Variation in daSP with difference in mean critical temperature (a) and mean event critical temperature (b).

Variability (r^2) explained by the difference in average critical temperature increases from 0.46 to 0.82 when precipitation events are considered in the analysis. The percentage (blue text) in the first (third) quadrant represents the fraction of UBPs with positive (negative) daSP with higher average buffer (urban) temperature than in the urban (buffer) region. Source data are provided in Source_data.xlsx, on the sheet called Figure_S8.

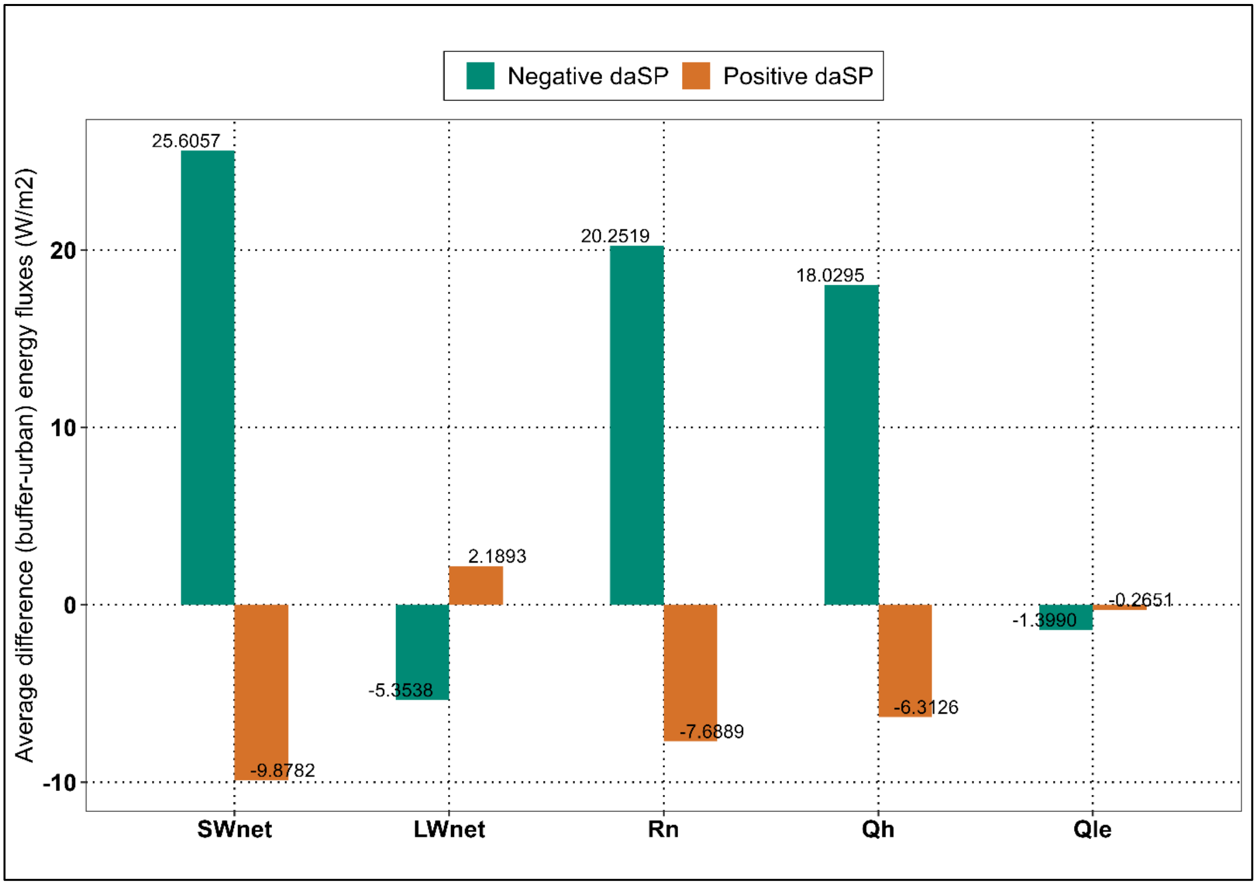

Figure S9: Average differences (buffer – urban) in the energy fluxes on the SDE50-days encountered by UBPs belonging to positive (orange) and negative (green) daSP suites. Source data are provided in Source_data.xlsx, on the sheet called Figure_S9.

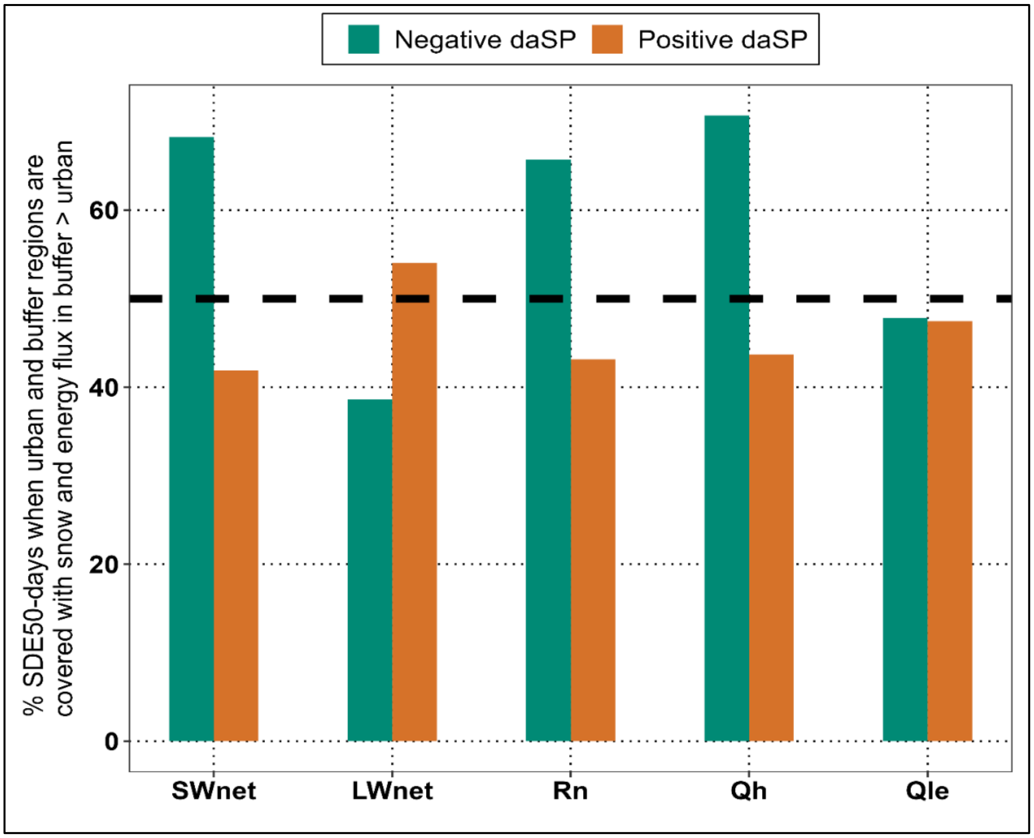

Figure S10: Similar to Figure 2b, for SDE50-days when urban and buffer regions are covered by snow. Source data are provided in Source_data.xlsx, on the sheet called Figure_S10.

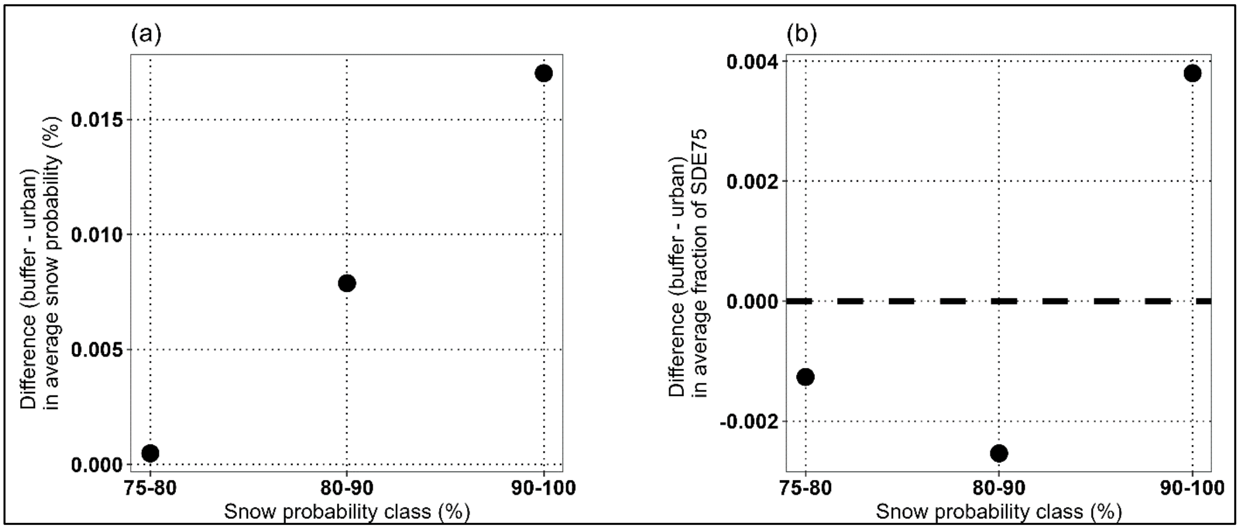

Figure S11: Variation in differences in average snow probability (a) and average fraction of frequency of snow-dominated events (SDE75) (b) encountered by all UBPs with respect to different snow probability ranges. Source data are provided in Source_data.xlsx, on the sheet called Figure_S11.

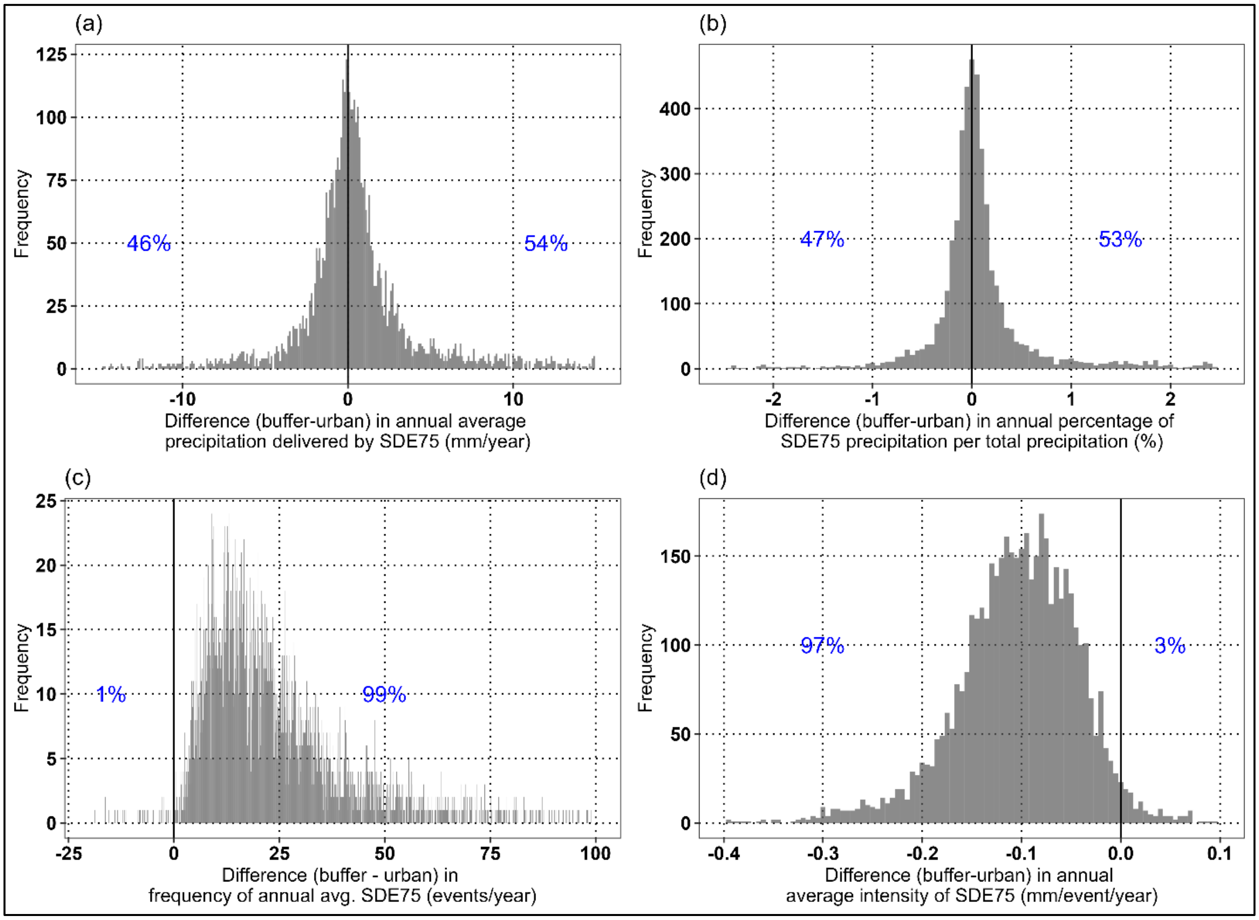

Figure S12: Similar to Figure 3, for SDE75. Source data are provided in Source_data.xlsx, on the sheet called Figure_S12.

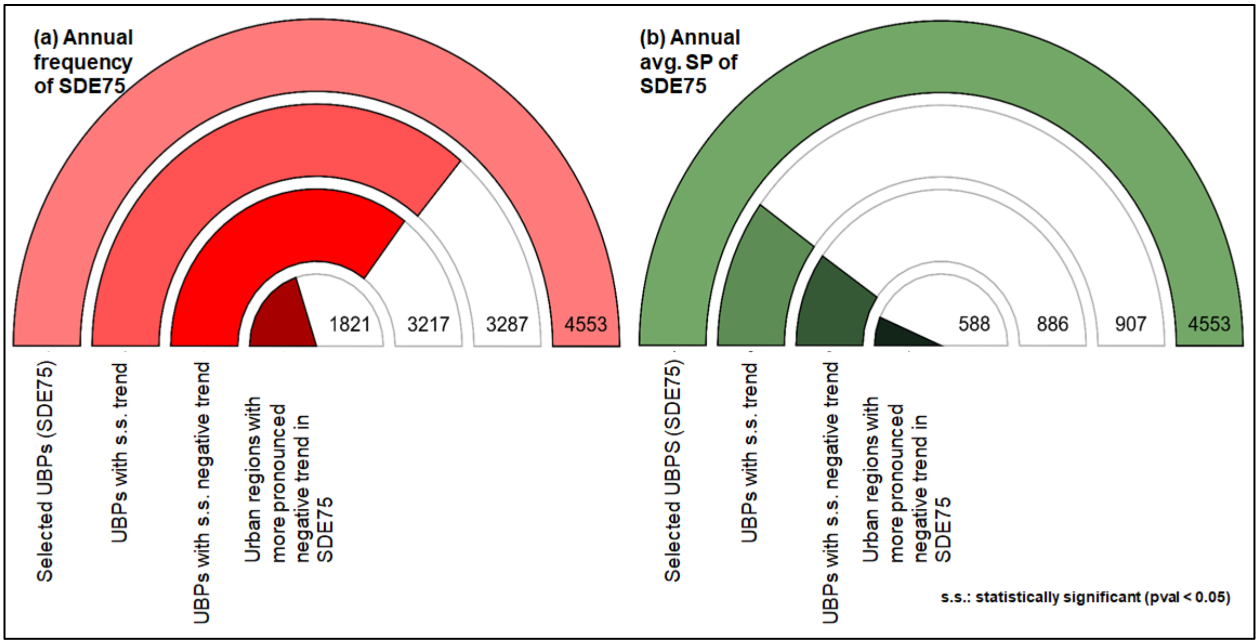

Figure S13: Similar to Figure 4, for SDE75. Source data are provided in Source_data.xlsx, on the sheet called Figure_S13_and_Table_S5.

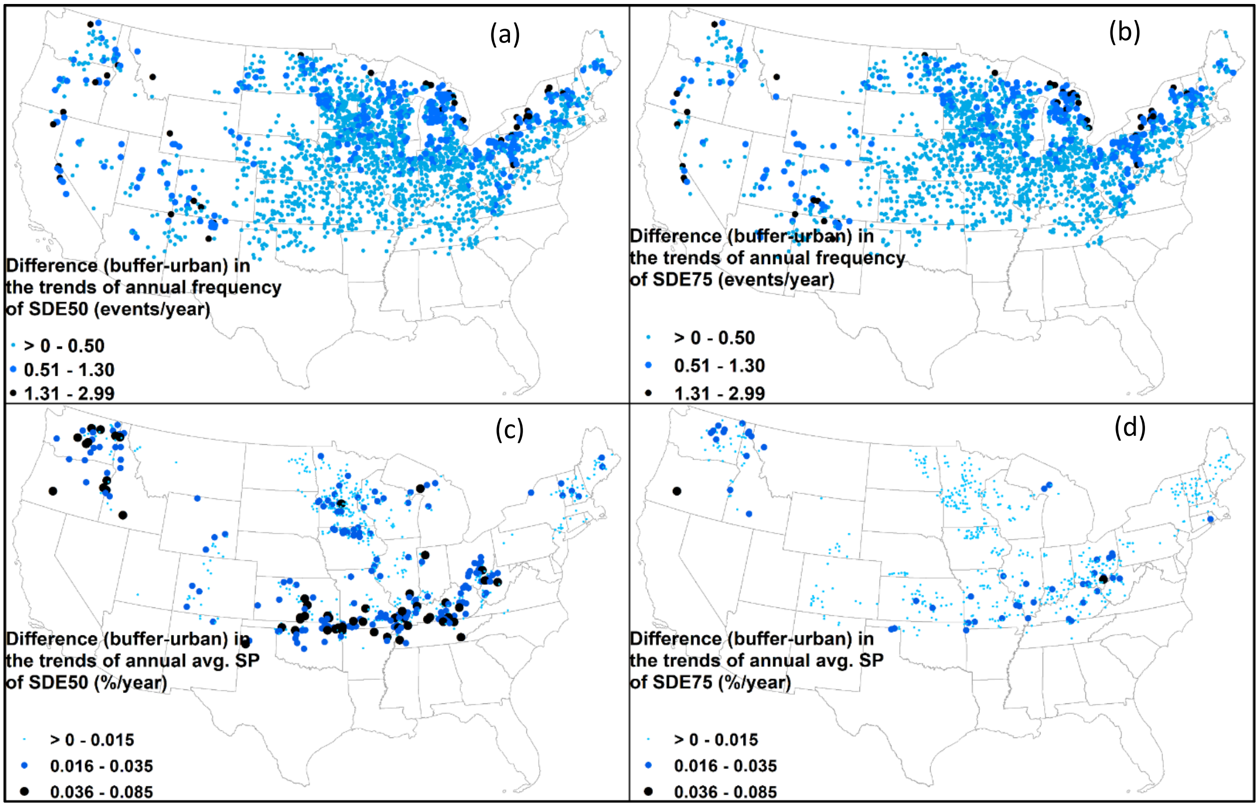

Figure S14: Geographic distribution of UBPs with difference (buffer – urban) in trends in 1) annual frequency of SDE50 (a), SDE75 (b), and annual average SP of SDE50 (c), SDE75 (d). Only the UBPs wherein both the regions exhibit statistically significant negative trends, and urban regions show a more pronounced negative trend w.r.t. its buffer counterpart are shown here. Source data are provided in Source_data.xlsx, on the sheet called Figure_4_and_Table_S5 and Figure_S13_and_Table_S5.

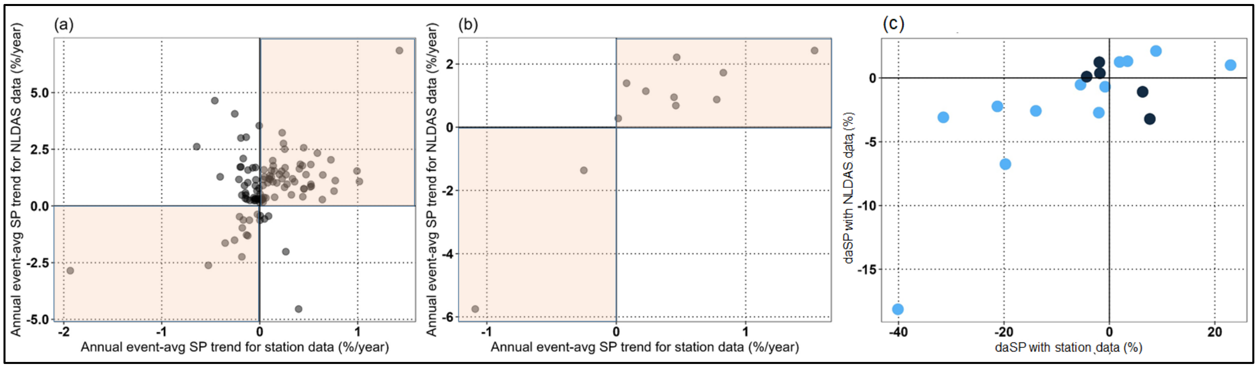

Figure S15: Comparison between station data and NLDAS data based on 1) the statistically significant trends of annual event-average SP for stations within urban (a) and buffer (b) regions; and 2) statistically significant daSP for UBPs with at least one station within urban and buffer regions each (c).

Comparison of statistically significant trends of annual event-avg. SP between 110 (11) stations that reside within the urban (buffer) areas and the nearest NLDAS grids (for the same duration as the data available at the individual stations) reveals that the trend directions of station and NLDAS annual event-average SP match for 70 stations (~64%) (a). In the case of buffer regions, the trend directions match for 100% of the stations. Overall, out of 121 stations, 81 stations (~67% stations) show trends in the same direction for both datasets (highlighted quadrants). Similar comparison based on the statistically significant daSP for 17 UBPs, where at least one station resides in either of the regions shows that for 12 stations out of 17 (~71%) the direction of daSP based on the station data matches with the daSP estimated with NLDAS data. Source data are provided in Source_data.xlsx, on the sheet called Figure_S15.

Supplementary tables

Table S1: Comparison of energy fluxes and albedo between urban and buffer regions on the days that receive SDE50 (SDE50-days). Source data are provided as a Source Data file.

Positive daSP		Values	Negative daSP		Values
**Part A)** For days when SDE50 are encountered by urban or buffer regions		(%)	**Part B)** For days when SDE50 are encountered by urban or buffer regions		(%)
1	urban SWnet > Buffer SWnet	60.22	1	Buffer SWnet > urban SWnet	63.68
2	urban Rn > Buffer Rn	60.63	2	Buffer Rn > urban Rn	65.08
3	urban Qh > Buffer Qh	59.02	3	Buffer Qh > urban Qh	66.73
4	urban albedo < Buffer albedo	62.21	4	Buffer albedo < urban albedo	71.74
5	urban albedo < Buffer albedo WHEN urban SWnet > Buffer SWnet	83.2	5	Buffer albedo < Urban albedo WHEN Buffer SWnet > urban SWnet	93.23
6	urban albedo < Buffer albedo WHEN urban Rn > Buffer Rn	80.83	6	Buffer albedo < Urban albedo WHEN Buffer Rn > urban Rn	89.72
7	urban albedo < Buffer albedo WHEN urban Qh > Buffer Qh	78.11	7	Buffer albedo < Urban albedo WHEN Buffer Qh > urban Qh	85.01
**Part C)** For days when SDE50 are encountered by urban or buffer regions, while both have snow-free ground			**Part D)** For days when SDE50 are encountered by urban or buffer regions, while both have snow-free ground		
1	urban SWnet > Buffer SWnet	60.93	1	Buffer SWnet > urban SWnet	47.42
2	urban Rn > Buffer Rn	63.53	2	Buffer Rn > urban Rn	57.23
3	urban Qh > Buffer Qh	59.94	3	Buffer Qh > urban Qh	49.68
4	urban albedo < Buffer albedo	66.96	4	Buffer albedo < urban albedo	62.32
5	urban albedo < Buffer albedo WHEN urban SWnet > Buffer SWnet	74.83	5	Buffer albedo < Urban albedo WHEN Buffer SWnet > urban SWnet	77.45
6	urban albedo < Buffer albedo WHEN urban Rn > Buffer Rn	75.97	6	Buffer albedo < Urban albedo WHEN Buffer Rn > urban Rn	75.11
7	urban albedo < Buffer albedo WHEN urban Qh > Buffer Qh	77.69	7	Buffer albedo < Urban albedo WHEN Buffer Qh > urban Qh	75.62

Table S2: Comparison of energy fluxes and albedo between urban and buffer regions on the SDE50-days, conditioned on sensible heat flux differences. Source data are provided as a Source Data file.

Positive daSP		Values (%)	Negative daSP		Values (%)
**Part A)** For days when SDE50 are encountered by urban or buffer regions			**Part B)** For days when SDE50 are encountered by urban or buffer regions		
1	urban SWnet > buffer SWnet WHEN urban Qh > buffer Qh	78.27	1	buffer SWnet > urban SWnet WHEN buffer Qh > urban Qh	79.59
2	urban Rn > buffer Rn WHEN urban Qh > buffer Qh	78.64	2	buffer Rn > urban Rn WHEN buffer Qh > urban Qh	80.28
3	urban albedo < buffer albedo WHEN buffer Qh > urban Qh	78.11	3	buffer albedo < urban albedo WHEN buffer Qh > urban Qh	85.01
**Part C)** For days when SDE50 are encountered by urban or buffer regions, while both have snow-free ground			**Part D)** For days when SDE50 are encountered by urban or buffer regions, while both have snow-free ground		
1	urban SWnet > buffer SWnet WHEN urban Qh > buffer Qh	79.55	1	buffer SWnet > urban SWnet WHEN buffer Qh > urban Qh	70.79
2	urban Rn > buffer Rn WHEN urban Qh > buffer Qh	80.21	2	buffer Rn > urban Rn WHEN buffer Qh > urban Qh	79.07
3	urban albedo < buffer albedo WHEN buffer Qh > urban Qh	77.69	3	buffer albedo < urban albedo WHEN buffer Qh > urban Qh	75.62

Table S3: Comparison of energy fluxes based on fraction (%) of SDE50-days when both the regions are covered with snow (termed as snow-covered days) i.e. albedo for both the regions >= 0.35. Source data are provided as a Source Data file.

Positive daSP		Values	Negative daSP		Values
**Part A)** For days when SDE50 are encountered by snow-covered urban and buffer regions		(%)	**Part B)** For days when SDE50 are encountered by snow-covered urban and buffer regions		(%)
1	urban albedo < Buffer albedo WHEN urban SWnet > Buffer SWnet	87.61	1	Buffer albedo < Urban albedo WHEN Buffer SWnet > urban SWnet	96.44
2	urban albedo < Buffer albedo WHEN urban Rn > Buffer Rn	82.36	2	Buffer albedo < Urban albedo WHEN Buffer Rn > urban Rn	93.01
3	urban albedo < Buffer albedo WHEN urban Qh > Buffer Qh	75.79	3	Buffer albedo < Urban albedo WHEN Buffer Qh > urban Qh	85.73
4	urban SWnet > buffer SWnet WHEN urban Qh > buffer Qh	76.2	4	buffer SWnet > urban SWnet WHEN buffer Qh > urban Qh	81.15
5	urban Rn > buffer Rn WHEN urban Qh > buffer Qh	75.72	5	buffer Rn > urban Rn WHEN buffer Qh > urban Qh	78.64
6	urban albedo < buffer albedo WHEN urban Qh > buffer Qh	75.79	6	buffer albedo < urban albedo WHEN buffer Qh > urban Qh	85.73

Table S4: Mean land cover fraction for buffer regions belonging to positive and negative daSP. Source data are provided as a Source Data file.

**Land covers**	**Fraction of land cover (%)**	
	**Positive daSP**	**Negative daSP**
Water	3.942	3.265
Developed	4.418	2.375
Barren	0.449	1.073
Deciduous	20.288	9.144
Evergreen	10.257	28.250
Mixed	6.682	4.185
Shrub	11.824	25.160
Grassland	5.022	11.068
Hay	8.209	3.219
Crops	22.481	9.168
Woody Wetlands	5.004	2.130
Herbaceous Wetlands	1.422	0.891

Table S5: Climate variables steering the spatial distribution of UBPs with higher magnitude of negative urban trend as compared to the buffer. Source data are provided as a Source Data file.

Variable for trend calculation	Conditions based on trend directions of urban and buffer regions	No. of UBPs satisfying the condition for SDE50	No. of UBPs satisfying the condition for SDE75
Annual frequency of snow dominated events	**Population:** UBPs where both the regions show negative trend of annual frequency of snow-dominated events and urban trend is more negative	1991<br>(Part A)	1821<br>(Part B)
	**Out of population,** UBPs where both the regions show positive trend of annual average temperature and urban trend is more positive	793 (40%)	726 (40%)
	**Out of population,** UBPs where both the regions show negative trend of annual frequency of precipitation events and urban trend is more negative	1908 (96%)	1734 (95%)
	**Out of population**<br>(UBPs where both the regions show positive trend of annual average temperature and urban trend is more positive)<br>**OR**<br>(UBPs where both the regions show negative trend of annual frequency of precipitation events and urban trend is more negative)	1938 (97%)	1764 (97%)
Annual average snow probability of snow dominated events	**Population:** UBPs where both the regions show negative trend of annual avg. snow probability of snow-dominated events and urban trend is more negative	675<br>(Part C)	588<br>(Part D)
	**Out of population,** UBPs where both the regions show positive trend of annual average temperature and urban trend is more positive	230 (34%)	221 (38%)
	**Out of population,** UBPs where both the regions show positive trend of annual average critical event temperature and urban trend is more positive	503 (75%)	411 (70%)

	**Out of population**<br>(UBPs where both the regions show positive trend of annual average temperature and urban trend is more positive)<br>**OR**<br>(UBPs where both the regions show positive trend of annual average critical event temperature and urban trend is more positive)	562 (83%)	484 (82%)
